# Decreased Transition Rate From Situational Insomnia to Chronic Insomnia by One-Week Internet Cognitive Behavioral Treatments for Insomnia During the COVID-19 Pandemic

**DOI:** 10.3389/fpsyt.2022.837399

**Published:** 2022-03-22

**Authors:** Fei Feng, Chenxi Zhang, Hanwen Liang, Guojian Xu, Xian Luo, Shuai Liu, Yan Xu, Lulu Yang, Li Zhang, Lianhong Lin, Bin Zhang

**Affiliations:** ^1^Department of Psychiatry, Nanfang Hospital, Southern Medical University, Guangzhou, China; ^2^Guangdong-Hong Kong-Macao Greater Bay Area Center for Brain Science and Brain-Inspired Intelligence, Southern Medical University, Guangzhou, China; ^3^Shenzhen Kangning Hospital, Shenzhen, China; ^4^Guangdong Mental Health Center, Guangdong Provincial People's Hospital, Guangdong Academy of Medical Sciences, Guangzhou, China

**Keywords:** CBTI, insomnia severity index (ISI), pre-sleep arousal scale (PSAS), hospital anxiety and depression scale (HADS), hyperarousal, COVID-19

## Abstract

**Purpose:**

The purpose of the study was to determine the long-term effects of one-week self-guided internet cognitive behavioral treatments for insomnia (CBTI) on situational insomnia during the COVID-19 pandemic.

**Patients and Methods:**

The participants with situational insomnia (*n* = 194) were recruited from March 2020 to April 2020 in Guangzhou, China. The insomnia severity index (ISI), pre-sleep arousal scale (PSAS), and hospital anxiety and depression scale (HADS) were evaluated at baseline and a one-week internet CBTI program was delivered to all individuals. The participants were divided into the complete treatment group (the participants completed all seven modules of the CBTI course, *n* = 75), and the incomplete treatment group (the participants completed 0–6 modules of the CBTI course, *n* = 119). A total of 135 participants completed the post-intervention assessments. At 3 months follow-up, a total of 117 participants (complete treatment group: *n* = 51; incomplete treatment group: *n* = 66) completed the assessments of the ISI, PSAS and HADS. The transition rate from situational insomnia to chronic insomnia (duration of insomnia ≥ 3 months and ISI ≥ 8) was calculated in the two groups. Linear mixed effect model was used to investigate the effect of group (between the two groups), time (baseline vs. follow-up), and interaction (group x time) on various questionnaire score.

**Results:**

The transition rate from situational insomnia to chronic insomnia was significantly lower in the complete treatment group compared to the incomplete treatment group (27.5%, 14/51 vs. 48.5%, 32/66, *p* = 0.023). There were significant differences in group effect (*p* = 0.032), time effect (*p* = 0.000) and group × time effect (*p* = 0.048) between the two groups in the ISI total score. The ISI total scores decreased in both groups during follow-up compared to their baseline values, with a greater magnitude of decrease in the complete treatment group. There were no significant group x time effects between the two groups in the PSAS-total score, PSAS-somatic, PSAS-cognitive score, HADS total score, HADS anxiety score or HADS depression score.

**Conclusion:**

Our results suggested that one-week self-guided internet CBTI prevented the development of chronic insomnia from situational insomnia during the COVID-19 pandemic.

## Introduction

Situational insomnia, a form of primary insomnia according to Diagnostic and Statistical Manual of Mental Disorders (DSM-5), is a common health problem (1). Situational insomnia usually lasts no more than a month (e.g., a few days or a few weeks) and is often related to specific stressful circumstances, such as life events or rapid changes in sleep schedules or environment, or use of stimulants such as caffeine. Most people have experienced situational insomnia, especially in response to situational stress (2). Situational insomnia usually resolves when the initial stressful event or situation is settled. However, poor sleep in unusual circumstances may also depend upon individual sensitivity to the stress (3). For example, for individuals who are more vulnerable to sleep disturbances, insomnia may persist over time long after the resolution of the initial stressful event and become chronic.

The Corona Virus Disease 2019 (COVID-19) is by far the largest outbreak of atypical pneumonia since the severe acute respiratory syndrome (SARS) outbreak in 2003. Within weeks of the initial outbreak the total number of cases and deaths exceeded those of SARS (4), which created enormous stress for the public. The COVID-19 pandemic and its societal consequences of mass home confinement created a stressful situation for many people across the globe. Being forced to stay at home, work from home, do home-schooling with children, drastically minimize outings, and reduce social interaction, could have major impacts on sleep (5). It has been shown that there is a significant increase in the incidence of insomnia during the COVID-19 pandemic (6). Sleep plays a fundamental role in both mental and physical health. For instance, sleep is involved in emotion regulation (7) and immune functions (8). Chronic insomnia and prolonged sleep loss increase risks of long-term adverse consequences for mental, physical, and occupational health (9). Therefore, the individuals who experienced significant sleep disturbances during the COVID-19 pandemic may be at greater risk for the long-term adverse health outcomes. Thus, protecting sleep during this pandemic is particularly important to build resilience and cope more effectively with the social confinement, distress, and uncertainty.

Around the world, COVID-19 epidemic continues to spread, creating significant disturbances in people's sleep and potentially triggering more situational insomnia. The COVID-19 pandemic seems to be a precipitating factor for the development of situational insomnia in a stressful atmosphere (10). The occurrence and course of situational insomnia are associated with the risk of developing chronic insomnia (11). Ellis et al. reported that about 40% of the patients with situational insomnia eventually developed chronic insomnia (12, 13). Therefore, it is necessary to seek effective intervention methods to prevent the development of chronic insomnia from situational insomnia.

Cognitive behavioral treatment for insomnia (CBTI) is currently considered the preferred treatment for insomnia (14). It has been shown that the internet CBTI treatment significantly reduced sleep latency and improved insomnia symptoms. The improvement in sleep lasted as long as 1 year after treatment (15). Recent evidence shows that CBTI can be used to treat sudden-onset (acute) insomnia due to rapid stress-induced situation changes (16, 17). For example, CBTI has been shown to improve sleep in patients with post-traumatic stress disorder (PTSD) and the effects lasted for at least 6 months (18). We previously demonstrated that one-week internet CBTI resulted in an immediate improvement of situational insomnia and pre-sleep somatic hyperarousal in the adults recruited from the community during the COVID-19 pandemic (19). We hypothesized that one-week internet CBTI could prevent the development of chronic insomnia from situational insomnia. In this study we performed self-guided one-week internet CBTI to the adults with situational insomnia in the community during the COVID-19 pandemic. The long-term effects of CBTI on insomnia, pre-sleep arousal, anxious and depressive symptoms were determined at 3 months after the intervention.

## Materials and Methods

### Participants and Procedures

**Figure 1:**
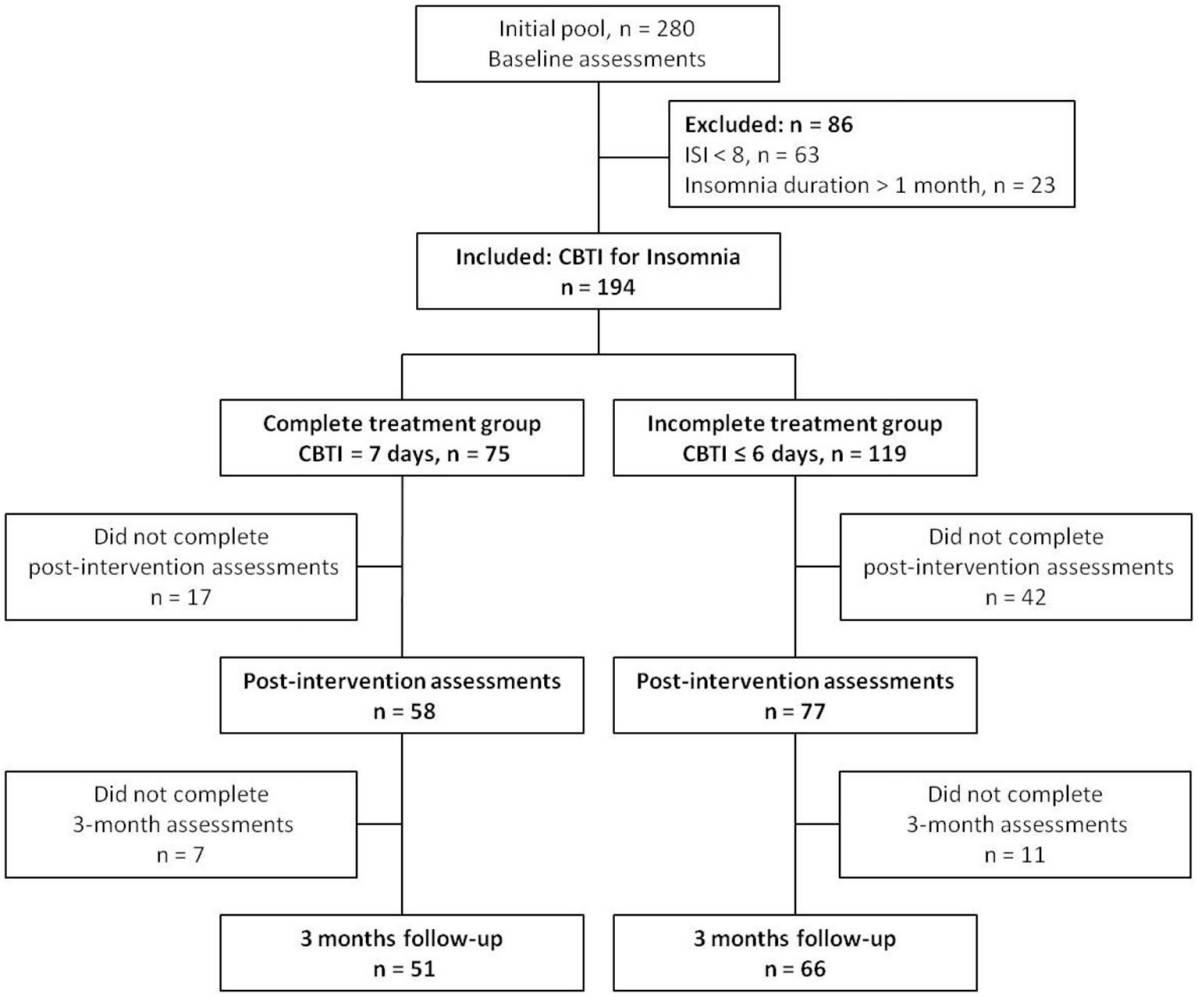
Flow chart of the study.

The study protocol was approved by the Ethics Committee in Nanfang Hospital, Southern Medical University. Individuals with situational insomnia were recruited through a campaign named “The Prevention and Protection Handbook against Epidemic” sponsored by the local government of Guangzhou, China from March to April in 2020. The diagnosis of situational insomnia was based on the following criteria according to the DSM-5: (a) the individual had sleep-onset insomnia and/or sleep-maintenance insomnia for at least three nights a week (>30 min each time); (b) the duration of insomnia lasted no more than a month; (c) the sleep disturbance (or the associated daytime fatigue) caused significant distress or impairment in social, occupational or other areas of functioning; and (d) a, b and c occurred despite adequate opportunity for sleep. The inclusion criteria were as follows: (1) adults aged between 18 and 64 years, (2) individual was diagnosed as situational insomnia, and (3) individual had an insomnia severity index (ISI) score of 8 or more. The exclusion criteria were as follows: (1) individual who had insomnia for more than a month, and (2) the ISI score was <8. As shown in the flow chart (Figure 1), among the initial pool of participants (*n* = 280) recruited for the study, 63 participants were excluded because their ISI scores were <8, and 23 participants were excluded because they suffered from insomnia for more than a month.

The signed informed consent form was obtained from all participants prior to starting the study. The pre-test online questionnaire was collected when participants completed their registration, ISI, pre-sleep arousal scale (PSAS), hospital anxiety and depression scale (HADS) were assessed. A total of 194 participants completed baseline scale assessment. The 7-day internet CBTI course was delivered to all participants using a social media platform named Wechat. The CBTI course consisted seven modules (about 15 min each), including sleep hygiene education (day 1), sleep restriction (day 2), stimulation control (day 3), relaxation training (day 4), cognitive reconstruction (day 5), core thoughts about sleeping medicine (day 6), and summary and review (day 7) (19). After the first login, the system recorded the login history automatically. The participants were divided into complete treatment group (the participants completed all 7 modules of the CBTI course, *n* = 75), and incomplete treatment group (the participants completed 0–6 modules of the CBTI course, *n* = 119). Following 7 days of CBTI, the participants were asked to complete the second online questionnaire for the assessment of ISI, PSAS, and HADS within 1 week. A total of 135 completed the post-intervention online assessments, with 58 in the complete treatment group and 77 in the incomplete treatment group (*n* = 6, 10, 6, 9, 11, 21, and 14 for completion of 0, 1, 2, 3, 4, 5 and 6 modules of CBTI, respectively). At 3 months after the CBTI intervention, we sent out 3 reminders via Wechat within 1 week to remind all participants to submit the online assessments of ISI, PSAS and HADS. A total of 117 participants completed the 3 months follow-up assessments. The chronic insomnia was defined as (1) duration of insomnia symptoms ≥ 3 months, and (2) ISI ≥ 8. The frequency of insomnia symptoms was not considered in the definition of chronic insomnia. The transition rate from situational insomnia to chronic insomnia was calculated in the complete treatment and incomplete treatment groups.

### Measures

#### Insomnia Severity Index (ISI)

The ISI was used to evaluate the severity of insomnia. ISI consists of seven items. Each item is rated on a 5-point Likert scale (0 = none, 1 = mild, 2 = moderate, 3 = severe, 4 = extremely severe), with higher total scores indicating more severe insomnia. The score ranges for severity ratings for insomnia are: <8: no insomnia symptoms; 8–14: mild insomnia; 15–21: moderate insomnia; and 22–28: severe insomnia. ISI has adequate internal consistency and is a reliable self-report measure to evaluate perceived sleep difficulties. It can be used as a screening tool clinically or an outcome measure in insomnia treatment research (20). The ISI measures the initial, middle and late insomnia, sleep satisfaction, interference of insomnia with daytime functioning, noticeability of sleep problems by others, and distress about sleep difficulties. The ISI has adequate psychometric properties and sensitivity to detect insomnia and evaluate treatment response. Thorndike et al. validated an online ISI version and showed that the online ISI version and paper-and-pencil ISI version were comparable (21).

#### Pre-sleep Arousal Scale (PSAS)

The PSAS was used to evaluate pre-sleep arousal. The PSAS is composed of two subscales: cognitive arousal and somatic arousal, each contains 8 items. Each item is rated on a 5-point Likert scale that ranges from 1 (not at all) to 5 (extremely). The cognitive subscale describes cognitive arousal such as worry about falling asleep, being mentally alert at bedtime, and inability to shut off thoughts). The somatic subscale addresses physical arousal such as racing heart, muscle tension, and rapid breathing. The PSAS cognitive or somatic scores range from 8 to 40. High scores on both subscales indicate hyper arousal (22). The PSAS has been widely used as an effective screening tool for identifying sleep disturbances.

#### Hospital Anxiety and Depression Scale (HADS)

The HADS was used to assess the anxiety and depression symptoms. The HADS is a self-assessment questionnaire that focuses on the psychic symptoms of mood disorders. The HADS has seven items each for anxiety (HADS-A) and depression (HADS-D) subscales. HADS-A items focus on symptoms related to generalized anxiety, while HADS-D items focus on anhedonia symptoms, a central aspect of depression. Each item is rated on a 0–3 scale (ranging from 0 = no not at all, to 3 = yes definitely) with the total subscale score ranges from 0 to 21. Score ranges for severity ratings for depression or anxiety subscale are: 0–7 (Minimal), 8–10 (Mild), 11–13 (Moderate) and 14–21(Severe) (19).

### Statistical Analysis

All the analyses were performed using SPSS (version 22.0) and R (version 3.6.1) with package “lme4”. Demographic data and sleep patterns are presented as mean ± SD, percentage and interquartile range (IQR) as appropriate. The comparison between any two groups was analyzed using the Chi-square test or independent-samples *t*-test. Linear mixed effect model was used to investigate the effect of group (complete treatment vs. incomplete treatment), time (baseline vs. follow-up), and interaction (group x time) on various questionnaire score. In the linear mixed effect model, we put “subject” in the random effect and “time”, “group”, and “time x group” in the fixed effect with a varying intercept. Statistical significance was considered at level of *p* < 0.05.

## Results

### Demographic Characteristics and Sleep Patterns

The demographic data of the subjects of the complete treatment and incomplete treatment groups are presented in Table 1. The mean age was 37.5 years and 36.7 years for the complete treatment group and incomplete treatment group, respectively. The body mass index (BMI) was in the normal range (average 21.9 kg/m^2^) in both groups. The gender composition was similar in the two groups; the percentage of females was above 70% in both groups. There were no statistically significant differences between the two groups in education level, marital status, with whom the subject lives, employment status and monthly income.

**Table 1:** Demographic data.

	**Complete**	**Incomplete**	**t or χ^2^**	***p***
	**treatment**	**treatment**	**value**	**value**
	**(*n =* 58)**	**(*n =* 77)**		
Age (years)	37.5 ± 11.8	36.7 ± 11.6	t = 0.377	0.707
BMI (kg/m^2^)	21.9 ± 2.7	21.9 ± 3.3	t = −0.055	0.956
**Gender**			χ^2^ = 0.263	0.608
Male	15 (25.9)	23 (29.9)		
Female	43 (74.1)	54 (70.1)		
**Highest education level**			χ^2^ = 3.775	0.151
High school diploma or less	10 (17.3)	17 (22.1)		
Bachelor's degree	30 (51.7)	47 (61.0)		
Master's or doctoral degree	18 (31.0)	13 (16.9)		
**Marital status**			χ^2^ = 0.472	0.790
Single	20 (34.5)	31 (40.3)		
Married	34 (58.6)	41 (53.2)		
Divorced	4 (6.9)	5 (6.5)		
**With whom the subject lives**			χ^2^ = 1.694	0.638
Alone	12 (20.7)	22 (28.6)		
Parents	20 (34.5)	23 (29.8)		
Child	23 (39.7)	26 (33.8)		
Friends	3 (5.1)	6 (7.8)		
**Employment status**			χ^2^ = 4.994	0.289
Full time	45 (77.6)	54 (70.1)		
Part time	3 (5.2)	1 (1.3)		
Unemployed	4 (6.9)	5 (6.5)		
Retired	2 (3.4)	9 (11.7)		
Student	4 (6.9)	8 (10.4)		
**Monthly income (**¥**)**			χ^2^ = 4.242	0.236
<3,000	7 (12.1)	12 (15.6)		
3,000–5,000	13 (22.4)	28 (36.4)		
5,000–10,000	18 (31.0)	17 (22.0)		
>10,000	20 (34.5)	20 (26.0)		

*Data are mean ± SD or N (%); BMI, body mass index; ¥, Chinese Yuan*.

There were no statistically significant differences between the complete treatment and incomplete treatment groups in the frequency of using tea, coffee, alcohol, cigarette in the previous year (Table 2). Most of the participants (67–80%) in the two groups never or seldom (1–3 times/week) drank tea or coffee, and over 90% of the participants in both groups did not or seldom drink alcohol in the past year. Only 3.4% and 7.8% of the participants in the complete treatment group and incomplete treatment group, respectively, smoked more than 3 times a week in the past year.

**Table 2:** Uses of tea, coffee, alcohol and cigarettes in the past year.

	**Complete**	**Incomplete**	**χ^2^**	***p***
	**treatment**	**treatment**	**value**	**value**
	**(*n =* 58)**	**(*n =* 77)**		
	**N (%)**	**N (%)**		
**Tea**			1.182	0.554
Never	12 (20.7)	16 (20.8)		
Seldom (1–3 times/week)	27 (46.5)	42 (54.5)		
Often (>3 times/week)	19 (32.8)	19 (24.7)		
**Coffee**			1.245	0.537
Never	19 (32.7)	32 (41.5)		
Seldom (1–3 times/week)	28 (42.3)	34 (44.2)		
Often (>3 times/week)	11 (19.0)	11 (14.3)		
**Alcohol**			1.579	0.454
Never	20 (34.5)	30 (39.0)		
Seldom (1–3 times/week)	36 (62.1)	41 (53.2)		
Often (>3 times/week)	2 (3.4)	6 (7.8)		
**Cigarettes**			1.672	0.383
Never	47 (81.0)	63 (81.8)		
Seldom (1–3 times/week)	9 (15.5)	8 (10.4)		
Often (>3 times/week)	2 (3.5)	6 (7.8)		

The sleep patterns of the complete treatment and incomplete treatment groups at baseline are presented in Table 3. The two groups had similar going to bed time and wake up time for weekdays and weekends. There were no statistically significant differences in daytime nap time between the two groups. Significant difference (*p* = 0.035) was observed between the two groups in the sleep latency. The sleep latency was >60 min in 22.4% and 41.5% of the participants in the complete treatment group and incomplete treatment group, respectively. The average sleep duration was 6.5 h in both groups during the COVID-19 outbreak. There were no statistically significant differences between the two groups in sleep duration.

**Table 3:** Sleep pattern.

	**Complete**	**Incomplete**	**t or χ^2^**	***p***
	**treatment**	**treatment**	**value**	**value**
	**(*n =* 58)**	**(*n =* 77)**		
**Time to go to bed [median (IQR)]**				
Weekdays	22:00	22:30	t = 0.436	0.664
	(00:56–23:05)	(01:52–23:30)		
Weekends	22:02	22:35	t = 0.216	0.830
	(01:00–23:29)	(02:00–23:30)		
**Time to wake [median (IQR)]**				
Weekdays	07:15	07:40	t = −1.001	0.316
	(06:37–09:15)	(07:00–08:52)		
Weekends	08:45	08:30	t = 0.973	0.331
	(07:30–10:00)	(07:30–10:15)		
Daytime nap	24.66 ± 29.70	24.48 ± 29.83	t = 0.034	0.973
(minutes, mean ± SD)				
**Sleep latency [minutes, N (%)]**			χ^2^ = 8.401	0.035^*^
<10	3 (5.2)	5 (6.5)		
11–30	17 (29.3)	23 (29.9)		
31–60	25 (43.1)	17 (22.1)		
>60	13 (22.4)	32 (41.5)		
Sleep duration	6.52 ± 3.55	6.52 ± 2.78	t = 0.011	0.992
(hours/day, mean ± SD)				

* *Indicates significance between the complete treatment group and the incomplete treatment group (p < 0.05, t - test). IQR, interquartile range*.

### Effect of CBTI on Insomnia

**Figure 2:**
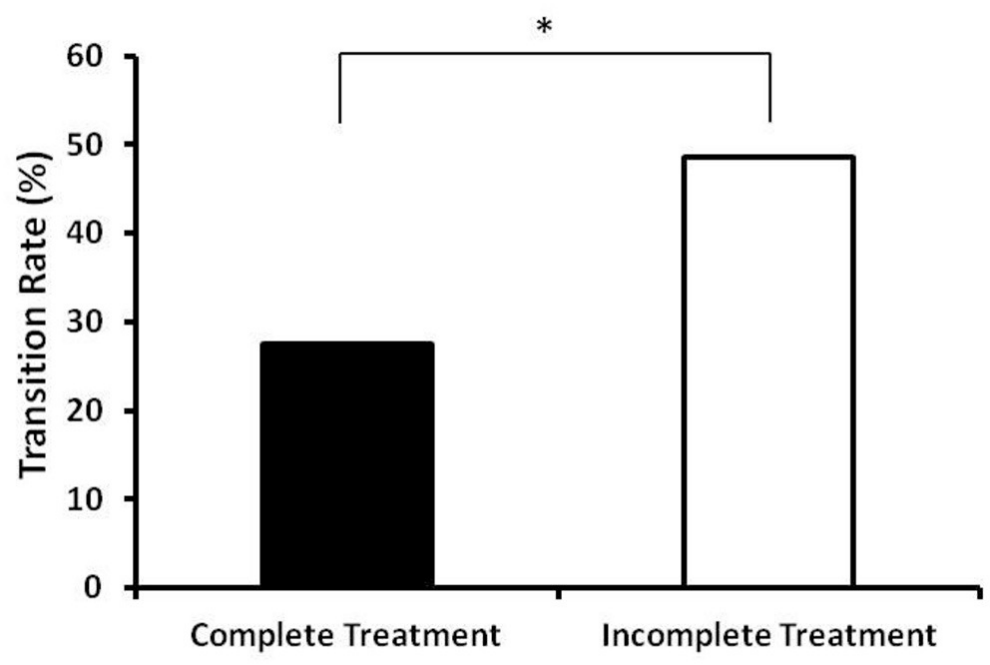
Transition rates from situational insomnia to chronic insomia in the complete treatment group and incomplete treatment group. The complete treatment group had a significantly lower transition rate compare to the incomplete treatment group (27.5%, 14/51 vs. 48.5%, 32/66). *Indicates significant differences between the two groups (*p* < 0.05).

The transition rates from situational insomnia to chronic insomnia are presented in Figure 2. The transition rates were significantly lower (*p* = 0.023) in the complete treatment group (27.5%, 14/51) compared to the incomplete treatment group (48.5%, 32/66).

**Figure 3:**
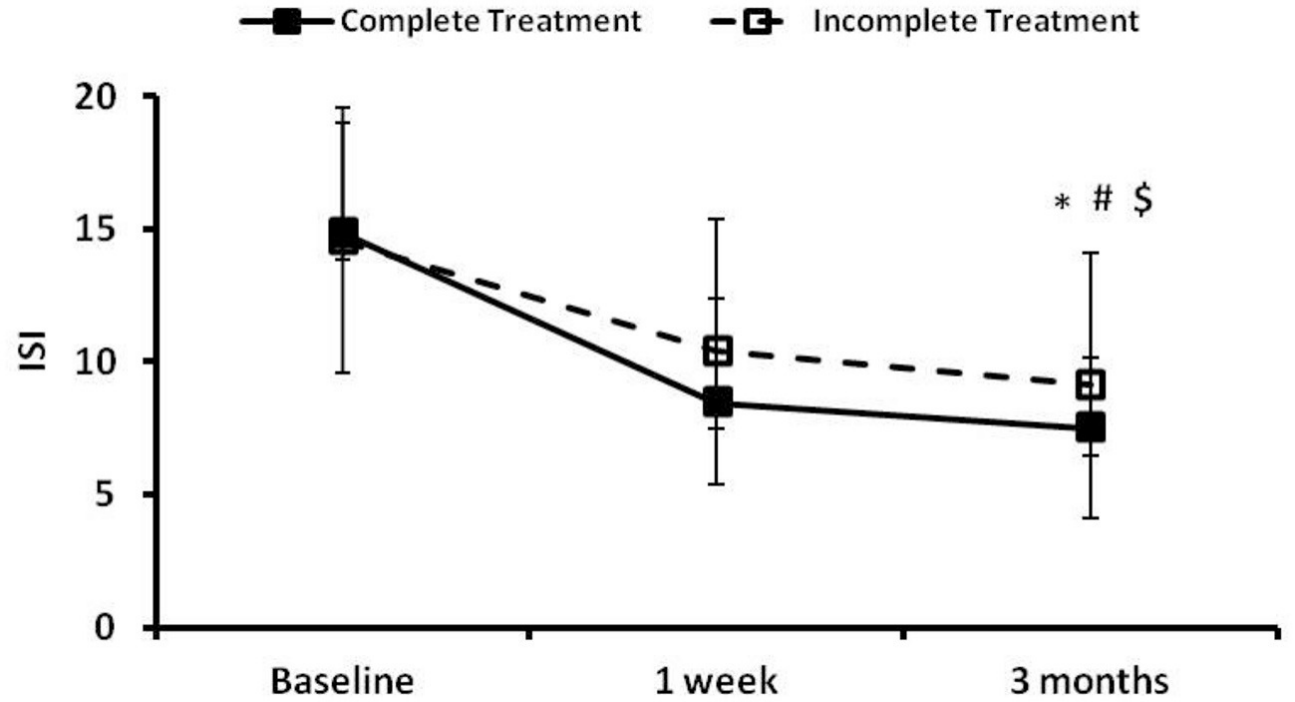
The insomnia severity index (ISI) at the preintervention baseline, and 1 week and 3 months following cognitive behavioral treatments for insomnia (CBTI). *, #, and $ indicate significant differences (*p* < 0.05) for the time effect, group effect and group × time effect, respectively. Complete treatment group, *n* = 58; incomplete treatment group, *n* = 77.

The time courses of ISI in the complete treatment and the incomplete treatment groups are presented in Figure 3. There were no significant differences between the two groups in ISI at baseline. There were significant time effect (*p* = 0.000), group effect (*p* = 0.032) and group × time effect (*p* = 0.048) between the two groups in ISI at 3 months follow-up. The ISI total scores decreased in both groups during follow up compared to their baseline values, with a greater magnitude of decrease in the complete treatment group (Figure 3).

### Effect of CBTI on the PSAS

**Figure 4:**
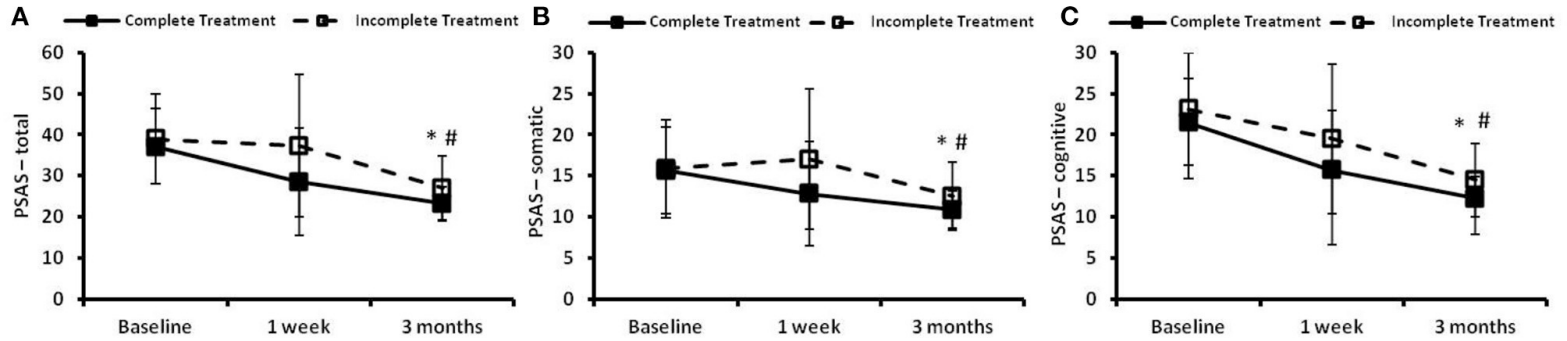
The pre-sleep arousal scale (PSAS) at the preintervention baseline, and 1 week and 3 months following cognitive behavioral treatments for insomnia (CBTI). **(A)** PSAS-total; **(B)** PSAS-somatic; **(C)** PSAS-cognitive. * and # indicate significant differences (*p* < 0.05) for the time effect and group effect, respectively. Complete treatment group, *n* = 58; incomplete treatment group, *n* = 77.

The time courses of PSAS in the complete treatment and the incomplete treatment groups are presented in Figure 4. There were no significant differences in PSAS-total score, PSAS-somatic score or PSAS-cognitive scores between the two groups at baseline. Significant time effects and group effects (*p* < 0.05) were found between the two groups in the PSAS-total score (Figure 4A), the PSAS-somatic score (Figure 4B) and cognitive score (Figure 4C). There were no significant group × time effects observed between the two groups in the PSAS-total score, PSAS-somatic score or PSAS-cognitive score.

### Effect of CBTI on the HADS

**Figure 5:**
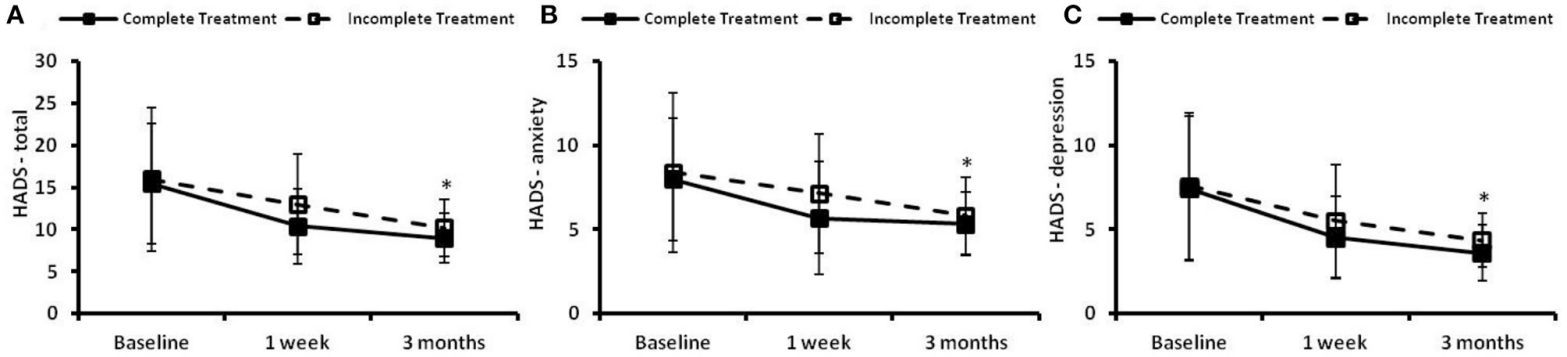
The hospital anxiety and depression scale (HADS) at the preintervention baseline, and 1 week and 3 months following cognitive behavioral treatments for insomnia (CBTI). **(A)** HADS–total; **(B)** HADS–anxiety; **(C)** HADS–depression. *Indicates significant differences (*p* < 0.05) for the time effect. Complete treatment group, *n* = 58; incomplete treatment group, *n* = 77.

The time courses of HADS in the complete treatment and the incomplete treatment groups are presented in Figure 5. There were no significant differences in the HADS-total, HADS-A or HADS-D scores at baseline between the two groups. Significant time effects (*p* < 0.05) between the two groups were found in the total score (Figure 5A), anxiety score (Figure 5B), and depression score (Figure 5C). There were no significant group effects and group × time effects observed between the two groups in the HADS-total score, HADS-anxiety score or HADS-depression score.

## Discussion

Our results showed that one-week internet CBTI program reduced insomnia at 1 week and 3 months follow-up. The CBTI also reduced the transition rate from situational insomnia to chronic insomnia at 3 months follow up. Our findings suggest that the one-week internet CBTI intervention result in both short-term and long-term improvement in sleep in individuals with situational insomnia during the COVID-19 pandemic. To our best knowledge, this is the first study to investigate the long-term effect of one-week internet CBTI on situational insomnia in the general population in the community during the COVID-19 pandemic.

CBTI is currently considered the preferred treatment to insomnia (14). Traditional CBTI requires face-to-face interviews. The treatment procedure is complex, time-consuming and costly. It typically requires patients to travel to the hospital/clinic for the face-to-face treatment, thus it may interfere with patients' routine work (23). The Internet CBTI has been paid more and more attention by experts and scholars these years, especially during the COVID-19 pandemic. Some professional committees recommend internet CBTI as the preferred method of intervention for insomnia (24).

The central principle of the CBTI is to target sleep-related dysfunctional thoughts, feelings, and behaviors and control the insomnia symptoms (25). Ellis and colleagues developed a “one-shot” CBTI intervention specifically to prevent the transition from acute insomnia to chronic insomnia (26). The “one-shot” CBTI includes a single 60- to 70-min session of CBTI, with an accompanying self-help pamphlet. The single session of CBTI has been shown to effectively reduce the transition rate from acute insomnia to chronic insomnia. It has been shown that there were no significant differences between the group treatment vs. the individualized treatment; both groups showed a remission rate of ~70% at 1-month follow-up (16). Randall evaluated the effects of “one-shot” CBTI on acute insomnia among prison inmates and showed a satisfactory remission rate at 1-month follow-up (17). Consistent with previous study (16), with similar pre-intervention ISI scores in the participants, we also observed similar remission rate (~73%) in the participants who completed the one-week CBTI at 3-month follow-up.

We have reported that one-week internet CBTI reduced pre-sleep somatic hyperarousal post-intervention (19). However, there were no statistically significant group × time effects observed between the complete treatment group and the incomplete treatment group in pre-sleep arousal at 3 months follow-up. People with hyperarousal trait were more likely to develop poor sleep during the COVID-19 (27). Effective interventions that target on hyperarousal were important in the treatment of insomnia. Hyperarousal refers to a broad pattern of excessive, poorly modulated responsiveness to stimuli during wakefulness (28). The 3P model of situational insomnia (predisposing, precipitating, and perpetuating) was proposed by Spielman and colleges in the 1980s (29). Hyperarusal can serve as a marker of predisposition or vulnerability to insomnia (29, 30). Hyperarousal is a key component in most prevailing etiological models of insomnia (31–33). People with cognitive hyperarousal trait have more dysfunctional beliefs about sleep (e.g., catastrophizing negative fallout of a poor night's sleep). Somatic hyperarousal (e.g., muscle tension) in bed presages poor sleep and is tied to reduced sleep efficiency and quality. Kalmbach et al. compared the intervention effects of CBTI and sleep restriction therapy (SRT) on menopausal insomniacs, showing that CBTI was more effective in reducing pre-sleep somatic hyperarousal and dysfunctional beliefs about sleep compared to the SRT in postmenopausal women (34). In a randomized controlled trial of 5 weeks of computerized cognitive behavioral therapy (cCBT), Vincent et al. showed that improvements in hyperarousal and time awake in bed partially mediated the impact of cCBT on sleep, and hyperarousal was a more significant mediator in explaining change associated with cCBT for insomnia (35).

In our study, there were no statistically significant differences (group × time effects) between the complete treatment group and the incomplete treatment group in improving anxiety and depressive symptoms. It has been shown that CBTI is beneficial for patients with residual depression and insomnia (36, 37). CBTI has been considered as an effective insomnia treatment for people with insomnia comorbid with depressive symptomatology (38). A randomized controlled study involving 1,149 participants with insomnia showed significantly better improvement in depression and anxiety symptoms through a self-help cognitive behavioral therapy program for insomnia (SHUTI) (39). However, CBTI has been shown to be more effective in treating insomnia than depression (38, 40). The inconsistency between our study and previous studies may be explained by following reasons. Firstly, the relaxation training of one-week CBTI was relatively short compared to the online CBTI treatment of 6 weeks or more (39, 41). Secondly, the program in our study only contained four relaxation audio pieces. Individual preferences for audio may affect the effectiveness of the treatment (42–44). A systematic review showed that progressive muscle relaxation training, music intervention and yoga were the most effective interventions for depression (43). Thirdly, our one-week internet CBTI program only used text and audio as a delivery method; it was possible that some of the participants did not understood all the details and key points of training.

There were some limitations in this study. Firstly, this was not a randomized controlled trial. The two groups were allocated based on how many days of CBTI the participants completed. We were comparing the effect of the complete treatment (7 days of CBTI) and the incomplete treatment (0–6 days of CBTI) on insomnia. Since the two groups were not randomly assigned, there may be selection bias and other confounding variables affecting the results. Secondly, the sample size in this study was relatively small. Only 60% of the participants (117 out of 194) completed the assessments at 3 months follow-up. Thirdly, all the questionnaires used for the assessments of ISI, PSAS and HADS were self-reported. The data may be prone to social desirability bias. Fourthly, the only follow-up duration in this study was 3 months. Although the current follow-up duration (3 months) allowed for establishing the diagnosis of chronic insomnia based on DSM5 criteria, long-term follow-up (e.g., 6 or 12 months) is needed for future study to examine whether the treatment effects of CBTI sustain over time.

In conclusion, our results showed that one-week internet CBTI program improved insomnia symptoms and reduced the transition rate from situational insomnia to chronic insomnia at 3 months follow-up, suggesting that one-week self-guided internet CBTI prevented the development of chronic insomnia from situational insomnia during the COVID-19 pandemic.

## Data Availability Statement

The original contributions presented in the study are included in the article/supplementary material, further inquiries can be directed to the corresponding author/s.

## Ethics Statement

The studies involving human participants were reviewed and approved by Ethics Committee of Nanfang Hospital of Southern Medical University. The patients/participants provided their written informed consent to participate in this study.

## Author Contributions

FF and BZ: conceptualization. SL, FF, CZ, and BZ: methodology. FF: writing–original draft. FF, CZ, HL, XL, LY, and GX: formal analysis. FF, HL, XL, YX, LZ, LL, LY, and GX: investigation and resources. CZ, SL, and BZ: writing–review and editing. GX, CZ, HL, LL, and LZ: data curation. XL, GX, and BZ: funding acquisition. BZ: supervision and project administration. All authors have approved the final manuscript.

## Funding

This work was supported by the National Natural Science Foundation of China (Grant No. 82071488 to BZ, Grant No. 81901348 to SL, and Grant No. 72101107 to XL), the President Foundation of Nanfang Hospital, Southern Medical University (Grant No. 2019Z014 to BZ), the Shenzhen Fund for Guangdong Provincial High-level Clinical Key Specialties (Grant No. SZGSP013 to GX), and the Shenzhen Key Medical Discipline Construction Fund (No. SZXK041).

## Conflict of Interest

The authors declare that the research was conducted in the absence of any commercial or financial relationships that could be construed as a potential conflict of interest.

## Publisher's Note

All claims expressed in this article are solely those of the authors and do not necessarily represent those of their affiliated organizations, or those of the publisher, the editors and the reviewers. Any product that may be evaluated in this article, or claim that may be made by its manufacturer, is not guaranteed or endorsed by the publisher.
